# Two‐Dimensional Perovskite‐Gated AlGaN/GaN High‐Electron‐Mobility‐Transistor for Neuromorphic Vision Sensor

**DOI:** 10.1002/advs.202202019

**Published:** 2022-07-22

**Authors:** Xitong Hong, Yulong Huang, Qianlei Tian, Sen Zhang, Chang Liu, Liming Wang, Kai Zhang, Jia Sun, Lei Liao, Xuming Zou

**Affiliations:** ^1^ Key Laboratory for Micro/Nano Optoelectronic Devices of Ministry of Education& Hunan Provincial Key Laboratory of Low‐Dimensional Structural Physics and Devices School of Physics and Electronics Hunan University Changsha 410082 P. R. China; ^2^ Hunan Key Laboratory for Super Microstructure and Ultrafast Process School of Physics and Electronics Central South University Changsha 410083 P. R. China; ^3^ Science and Technology on Monolithic Integrated Circuits and Modules Laboratory Nanjing 210016 P. R. China; ^4^ State Key Laboratory for Chemo/Biosensing and Chemometrics College of Semiconductors (College of Integrated Circuits) Hunan University Changsha 410082 P. R. China

**Keywords:** AlGaN/GaN, optoelectronic synapse, perovskites, vision sensors

## Abstract

The extraordinary optoelectronic properties and continued commercialization of GaN enable it a promising component for neuromorphic visual system (NVS). However, typical GaN‐based optoelectronic devices demonstrated to data only show temporary and unidirectional photoresponse in ultraviolet region, which is an insurmountable obstacle for construction of NVS in practical applications. Herein, an ultrasensitive visual sensor with phototransistor architecture consisting of AlGaN/GaN high‐electron‐mobility‐transistor (HEMT) and two‐dimensional Ruddlesden–Popper organic–inorganic halide perovskite (2D OIHP) is reported. Utilizing the significant variation in activation energy for ion transport in 2D OIHP (from 1.3 eV under dark to 0.4 eV under illumination), the sensor can efficiently perceive and storage optical information in ultraviolet–visible region. Meanwhile, the photo‐enhanced field‐effect mechanism in the depletion‐mode HEMT enables gate‐tunable negative and positive photoresponse, where some typical optoelectronic synaptic functions including inhibitory and excitatory postsynaptic current as well as paired‐pulse facilitation are demonstrated. More importantly, a NVS based on the proposed visual sensor array is constructed for achieving neuromorphic visual preprocessing with an improved color image recognition rate of 100%.

## Graphical abstract

This paper designs and fabricates a highly sensitive visual sensor based on two‐dimensional Ruddlesden–Popper organic–inorganic halide perovskite (2D OIHP)‐gated AlGaN/GaN high‐electron‐mobility‐transistor (HEMT). The photo‐enhanced field‐effect mechanism in the depletion‐mode HEMT enables gate‐tunable negative and positive photoresponse. Moreover, the HEMT‐based vision sensor array can be trained to perform color image recognition and preprocessing tasks with distinct kernels.

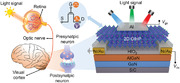

## 1 Introduction

Traditional vision systems with the von Neumann architecture are facing severe challenges because the physical separation of image sensing, memory and processing units causes an inherent cap on processing speed and operational efficiency.^[^ ^1^, ^2^ ^]^ In this circumstance, building neuromorphic visual system (NVS) with human brain‐inspired visual perception offers a promising way to solve the problem.^[^ ^3^, ^4^, ^5^, ^6^ ^]^ Compared to conventional machine visions, human vision operates by acquiring enormous visual information and simultaneously extracting distinctive features with high efficiency, which has attracted a large amount of attention in recent years as well as been the imitation object of researchers.^[^ ^7^, ^8^, ^9^, ^10^, ^11^, ^12^ ^]^ In order to achieve advanced NVS with intrinsic optical sensing and neuromorphic computing functions, the hardware implementation of visual sensors that include both efficient sensing and storage as well as preprocessing of photonic information over visible spectrum is necessary.^[^ ^8^ ^]^ Till now, memristors with resistive switching behavior have established promising paradigms in artificial neuromorphic devices.^[^ ^4^, ^12^, ^13^, ^14^, ^15^, ^16^, ^17^, ^18^, ^19^, ^20^ ^]^ However, the synaptic response in the two‐terminal memristive devices is normally obtained only by employing electric stimuli,^[^ ^21^, ^22^ ^]^ which is a daunting challenge for current NVS since the visual sensors require effective integration of light control over the devices electrical properties.

In comparison, the three‐terminal nonvolatile phototransistor‐geometry is regarded as a promising platform to accommodate diverse control parameters, which is beneficial for increasing device functionality.^[^ ^23^ ^]^ In such devices, the realization of biological functional behaviors relies on the synergy between conducting channel and neighboring gate component. Under illumination, one type photo‐excited carriers transport in the channel while another type carriers are trapped by localized states for a long period, leading to simultaneous sensing and storage from optical stimuli.^[^ ^24^ ^]^ Various high‐mobility semiconductors integrated optoelectronic synapses are responsible for excellent photon‐to‐electric conversion efficiency, including graphene,^[^ ^11^, ^25^ ^]^ carbon nanotubes,^[^ ^26^ ^]^ black phosphorus,^[^ ^27^, ^28^ ^]^ and so on. Compared with these materials, GaN‐based optoelectronic technologies have gone from exploratory research to industrial applications, which are particularly useful in ultraviolet photodetectors and short wavelength emitters.^[^ ^29^, ^30^, ^31^, ^32^ ^]^ Especially, the spontaneous and piezoelectric polarization effects cause high‐mobility two‐dimensional electron gas (2DEG) channel in AlGaN/GaN high‐electron‐mobility‐transistor (HEMT), resulting in large photogain (*G*) and low noise equivalent power at room temperature. However, limited by the unidirectional photoresponse, coupling of electrical and light stimulation is necessary for triggering inhibitory and excitatory postsynaptic current of these devices.^[^ ^29^, ^33^, ^34^ ^]^ For visual sensor applications, bidirectional light‐modulated synaptic behavior is highly desired for emulating the neurobiological functionality of bipolar cells in human retina. Furthermore, the data storage capacity in the HEMT‐based UV photodetectors, affected by the materials epitaxial quality and fabricated process, is still far less capable than the reported optoelectronic memories.^[^ ^34^ ^]^ Moreover, GaN absorbs only ultraviolet light efficiently as a direct wide bandgap semiconductor, which impedes the application of AlGaN/GaN HEMTs as visual sensors that require to be operated in visible region. As far as we know, HEMTs‐based NVS has not yet been reported.

In this work, we report, for the first time, the image sensing, memory as well as preprocessing functions can be achieved by employing a unique and simple combination of AlGaN/GaN HEMT with two‐dimensional Ruddlesden–Popper organic–inorganic halide perovskite (2D OIHP) (PEA)_2_PbI_4_, wherein PEA^+^ represents C_8_H_9_NH_3_^+^. Benefiting from short transit time for transporting electrons in the 2DEG channel and long lifetime of photo‐induced carriers at the 2D OIHP/HfO_2_ interface, an ultrahigh photoresponsivity (*R*) of 2.0 × 10^6^ A W^−1^ is demonstrated upon 532 nm laser illumination. As light power intensity (*P*_light_) increases from 0 to 15.1 µW cm^−2^, the activation energy (*E*_a_) for ion transport in 2D OIHP decreases significantly by a factor of three (from 1.3 to 0.4 eV), which provides a promising pathway for the imitation of visual memory. Meanwhile, the photo‐enhanced field‐effect mechanism enables gate‐tunable negative and positive photoresponses in AlGaN/GaN HEMTs. This property can be utilized to emulate typical synaptic functions, including excitatory postsynaptic current (EPSC), inhibitory postsynaptic current (IPSC), as well as paired‐pulse facilitation (PPF). Furthermore, we demonstrate that the HEMT‐based vision sensor array can be trained to perform color image recognition and preprocessing tasks with distinct kernels.

## 2 Results and Discussions

**Figure 1:**
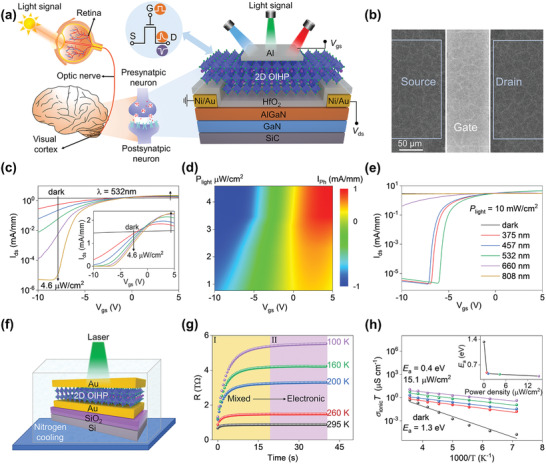
Design and characterization of the HEMT‐based visual sensor. a) Schematic illustration of a human visual system (left) composed of the HEMTs‐based visual sensor (right). b) SEM image of a HEMT‐based visual sensor. c) Transfer characteristic of the HEMT‐based visual sensor measured at *V*_ds_ = 0.1 V under various illumination intensity. The inset depicts the same curves in linear scale. d) *P*_light_ and *V*_gs_ dependent photocurrent extracted from the transfer characteristic curves shown in (c). e) Transfer characteristic curves of the proposed device under different wavelength. f) Vertical structural Au/(PEA)_2_PbI_4_/Au device with liquid nitrogen cooling system used in the experiment. g) Typical galvanostatic curves under 5 pA current and 15.1 µW cm^−2^ illumination at different temperature. h) The *σ*_ion_ *T*−1000/*T* plots of ionic conductivity under different laser power density. Solid lines are the fitting curves. The inset depicts the extracted *E*_a_ values.

The information processing model of human visual system is shown in **Figure** **1**a left, which is composed of optical system, retina and visual pathway. The light that carries visual information is projected onto retina through the optical system of eye, where the signals are integrated and transmitted to the visual cortex of brain. As shown in Figure 1a right, we mimic the perception of light in human eye and the synaptic connections in visual cortex by implementing visual sensor composed of AlGaN/GaN heterostructure and (PEA)_2_PbI_4_/HfO_2_ dielectric layer. Here, the 2D OIHP (PEA)_2_PbI_4_ is employed for high efficiency light absorption due to its advantages of high optical absorption coefficient, long carrier diffusion length, and excellent intrinsic ambient‐stability. Especially, the insulating PEA bilayer in 2D OIHPs causes an obvious light‐dependent ionic transport behavior, which is crucial for achievement of bidirectional light‐modulated synapse behavior in AlGaN/GaN HEMTs. The AlGaN/GaN heterostructure used in this article, typically for HEMT application, was grown on the SiC substrate by the metal‐organic chemical vapor deposition (MOCVD) system. After sputtering Ni/Au electrodes to form ohmic contacts, a 12‐nm HfO_2_ dielectric layer was deposited by atomic layer deposition (ALD) at 95 °C. Then, a 260‐nm 2D OIHP (PEA)_2_PbI_4_ film was formed by one‐step spin‐coating method for high efficient light harvesting, followed by the thermal evaporation of 20‐nm Al electrode. The scanning electron microscopy (SEM) image of the as‐deposited 2D OIHP film on HfO_2_ layer is shown in Figure S1a (Supporting Information), which exhibits compact polycrystalline texture with full surface coverage. The X‐ray diffraction (XRD) spectrum was also performed to investigate the crystallization characteristic^[^ ^35^ ^]^ (Figure S1b, Supporting Information). Figure 1b exhibits the SEM image of a typical device.

We first evaluate the photoresponse characteristics by focusing a 532 nm laser on the device. Figure 1c presents the transfer characteristic curves, that is, drain‐source current as a function of gate‐source voltage (*I*_ds_–*V*_gs_), measured at *V*_ds_ = 0.1 V under different laser power density (*P*_light_). Simultaneously, the inset depicts the same curves in linear scale. Apparently, under dark, the output current of the depletion mode AlGaN/GaN HEMT is almost independent of *V*_gs_, demonstrating a negligible field effect. In comparison, upon illumination, the 2DEG concentration modulation is greatly enhanced, inducing gate‐tunable negative and positive photocurrent (Figure 1d). The corresponding gate leakage is far below the output current (Figure S2, Supporting Information), suggesting that the photoresponse of our device mainly arises from the enhanced field effect. To assess the photoresponse to incident light intensity, the photoresponsivity is extracted with the equation:^[^ ^36^ ^]^

(1) $$R\;=\;\;\frac{\left|I_{\mathrm{light}}-\;I_{\mathrm{dark}}\right|}{P_{\mathrm{light}}\;S}=\frac{\left|I_{\mathrm{ph}}\right|}{P_{\mathrm{light}}\;S}$$

where *I*_dark_ and *I*_light_ refer to the output current with and without illumination, respectively, *S* denotes the active area of HEMT‐based visual sensor, and *I*_ph_ denotes the photocurrent. Here, the maximum *R* value is as high as 2.0 × 10^6^ A W^−1^ (Figure S3a, Supporting Information) upon 532 nm illumination, which demonstrates the capacity of HEMT‐based visual sensor for weak signal perception in visible region. Meanwhile, the dependence of *R* values on light intensity follows the power law, *R* ≈ *P*_light_^*α*^, where |*α*| is less than 1 (Figure S3b, Supporting Information). The non‐unity exponent is generally induced by the photogating effect.^[^ ^11^ ^]^ Here, to investigate the photoresponse mechanism, we also fabricated a control device without 2D OIHP layer (Figure S4, Supporting Information). The control device exhibits a high switch ratio exceeding 10^5^ under dark, while a positive photoresponse is observed under illumination. Meanwhile, the control device exhibits negligible photoresponse upon visible light illumination. In contrast, the 2D OIHP‐gated device exhibits a broadband photoresponse ranging from 375 to 808 nm (Figure 1e). Figure S1c (Supporting Information) illustrates the light absorption of 2D OIHP. Here, the broadband photoresponse beyond the absorption edge of the 2D OIHP can be attributed to the excitation of carriers from the valence band to the trap states within the perovskite bandgap, being similar to the extrinsic photoconductors based on conventional semiconductors.^[^ ^37^ ^]^ These results confirm that the 2D OIHP layer in our device is responsible for the photo‐enhanced field‐effect mechanism.

Upon illumination, the enhancement of field effect in 2DEG channel can thus be attributed to the accumulation of photo‐excited carriers at 2D OIHP/HfO_2_ interface, which causes an additional bias voltage at the interface and then changes the output current of AlGaN/GaN HEMT. The OIHPs have been demonstrated to be mixed electronic‐ionic conductors with ion vacancies (*V*_H_, *V*_I_, and *V*_Pb_, standing for H^+^, I^–^, and Pb^2+^ vacancies, respectively) as the main migration species.^[^ ^38^ ^]^ The ionic conductivity (*σ*_ion_) of (PEA)_2_PbI_4_ depends on the *E*_a_ for ion vacancies migration. To develop a clearer understanding of the photoresponse mechanism, we perform a quantitative assessment of the *E*_a_ values. As shown in Figure 1f, an Au/(PEA)_2_PbI_4_/Au vertical device was fabricated on silica substrate and measured under varying temperatures (*T*, 100–295 K). The mixed conductivity (*σ*_total_) of (PEA)_2_PbI_4_ film is composed of electronic conductivity (*σ*_e_) and ionic conductivity.^[^ ^39^ ^]^ Here, a common technique used in mixed conductor researches is employed to separate electronic conductivity from mixed conductivity. As a low current of 5 pA is switched on, the mobile ions in (PEA)_2_PbI_4_ channel are gradually depleted due to the ions accumulation at the upper and lower sides of the film, resulting in a decreasing ionic conductance (region I of Figure 1g). Once all the mobile ions are blocked at the boundaries, all that is left is the electronic conductivity; then, the channel conductance reaches a stable value (region II of Figure 1g). The mixed conductivity can be achieved with current–voltage fast‐scan measurements. Finally, the ionic conductivity at a wide range of temperature (100–280 K) and light intensity (0–15.1 µW cm^−2^) are extracted from the mixed conductivity: *σ*_ion_ = *σ*_total_ – *σ*_e_ (Figure S5, Supporting Information). The hopping‐like ionic transport behavior determined by energy barrier can be described with the following formula:^[^ ^40^ ^]^

(2) $$\begin{array}{ccc} \sigma_{\mathrm{ion}}T & = & ne\mu =\frac{Z_{i}e^{2}N_{A}C_{V0}D_{0}}{k_{B}V_{m}}\exp\;\left(-\frac{G_{v}}{5k_{B}T}-\frac{E_{a}}{k_{B}T}\right)\\ & = & \sigma_{0}\;\exp\left(\frac{E_{a}^{\mathrm{eff}}}{k_{B}T}\right) \end{array}$$

where *Z*_i_ denotes the ion charge, *C*_v_ denotes the intrinsic defects concentration, *N*_A_ denotes the Avogadro's constant, *D* denotes the diffusion coefficient, *k*_B_ denotes the Boltzmann's constant, *V*_m_ is the molar volume of 2D OIHP, *G*_v_ denotes the formation energy of vacancy defects, and $E_{a}^{\mathrm{eff}}$ is defined to consider excess vacancy formation energy in the vacancy‐mediated mechanism. To extract the *E*_a_ value, ln(*σ*_ion_ *T*) is plotted versus 1000/*T* (Figure 1h). Here, a linear region ranging from 140 to 280 K is detected, in which the corresponding slopes dramatically decrease with the increase of laser power, indicating the attenuation of the *E*_a_ value. Figure 1h inset depicts the activation energy of (PEA)_2_PbI_4_ with different light intensity. Obviously, as the light intensity increases from 0 to 15.1 µW cm^−2^, the ion activation energy decreases from 1.3 to 0.4 eV, consistent with the results of first‐principle calculation.^[^ ^41^, ^42^ ^]^ Accordingly, the photo‐excited mobile ions and electron–hole pairs increase the conductivity of 2D OIHP and thus lead to enhanced electric field at the 2DEG channel in the presence of gate voltage. In comparison, when the device is operated under dark, the insulating PEA bilayer causes a much lower mixed conductivity and a negligible field effect.

**Figure 2:**
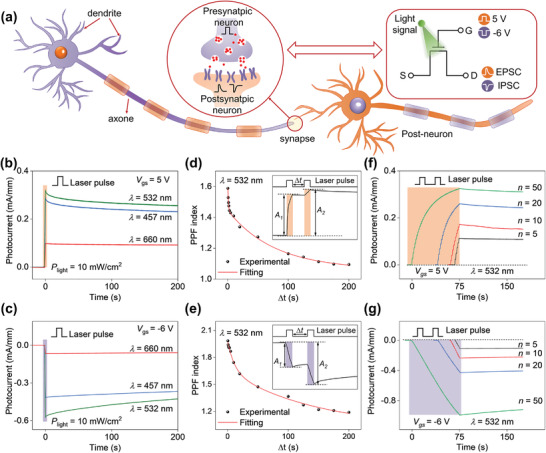
Gate‐tunable synaptic characteristics under optical stimulation of 532 nm. a) Schematic diagram of HEMTs‐based optical synapse with EPSC and IPSC behaviors. The photocurrent induced by 457/532/660 nm laser pulse (10 mW cm^−2^, 1 s) at b) *V*_gs_ = 5 V and c) *V*_gs_ = −6 V. PPF index as a function of Δ*t* triggered by the successive pulses (532 nm, 1 mW cm^−2^) under d) *V*_gs_ = 5 V and e) *V*_gs_ = −6 V, and the red curves refer to the exponentially fitting result. Insets: EPSC (d) and IPSC (e) of the device stimulated with two successive 532 nm laser pulses (Δ*t* = 0.2 s). Optical‐triggered current change of the device under 532 nm pulsed stimuli (*P*_light_ = 0.1 mW cm^−2^, duration: 0.5 s, interval: 1 s) at f) *V*_gs_ = 5 V and g) *V*_gs_ = −6 V.

The perceiving and processing of external information in the human brain mainly depend on a complicated neuron network, which is composed of abundant neurons and their gapped junctions named synapses. The connection strength between neurons is determined by recent activity of the synapses, which is called synaptic plasticity. Under stimulation, the synapses enable presynaptic neurons to deliver signals to postsynaptic neurons by neurotransmitter, resulting in the EPSC or IPSC. These postsynaptic current would be summed in postneurons, which determine the fire of neurons. In our HEMTs‐based visual sensor, the source/drain electrode corresponds to the anterior/posterior membranes of the biological synapses, and the light pulses are equivalent to the presynaptic action potential (**Figure** **2**a). As expected, the EPSC and IPSC are triggered by optical stimuli with light intensity of 10 mW cm^−2^ at *V*_gs_ = 5 V and *V*_gs_ = −6 V, respectively (Figure 2b,c). The PSC change (ΔPSC) increases as the incident light wavelengths vary from 660 nm (red) to 457 nm (blue) to 532 nm (green), which can be associated with the light absorption of 2D OIHP in visible region (Figure S1c, Supporting Information). After the laser is turned off, the EPSC and IPSC can effectively retain 73.2% and 52.2%, respectively, within the experimental time of 1200 s (Figure S6a,b, Supporting Information), suggesting an excellent nonvolatility of the device. These results are quite different from previously reported HEMT‐based photodetectors, which exhibit temporary and unidirectional photoresponse in ultraviolet region.^[^ ^34^ ^]^ Meanwhile, multiple cycles of programming and erasing processes ensure the operation stability of our device (Figure S6c,d, Supporting Information). Notably, the programmed current is hard to be completely erased by *V*_gs_ pulse. The recovery of the initial state relies on the combination of *V*_gs_ and light pulses (Figure S6e,f, Supporting Information), which can be understood by the light‐dependent ion motion in 2D OIHP (Figure S7, Supporting Information).

Next, paired‐pulse facilitation (PPF), referring to the reinforced postsynaptic response caused by two successive synaptic stimuli, is emulated based on the device. PPF is essential for recognizing and decoding visual signals in the biological neural system. To imitate this characteristic, two successive optical pulses (*λ* = 532 nm, duration = 500 ms, *P*_light_ = 1 mW cm^−2^) with time interval, Δ*t* = 1 s, were applied to the device. As shown in Figure 2d,e insets, the second spike causes higher *I*_ds_ amplitude (*A*_2_) than the first spike does (*A*_1_). The PPF index (*A*_2_/*A*_1_ × 100%) is closely related to the interval time, which can be fitted with a double stretching exponential function as follows,^[^ ^14^, ^43^ ^]^

(3) $$\mathrm{PPF}\;=\;1+\;C_{1}\exp\left(-\Delta t/\tau_{1}\right)+\;C_{2}\exp\left(-\Delta t/\tau_{2}\right)$$

where *C*_1_ and *C*_2_ denote the initial facilitation magnitudes of the rapid and slow facilitation states, and *τ*_1_ and *τ*_2_ denote the characteristic relaxation time of the rapid and slow facilitation states, respectively. Here, the fitted *τ*_1_ and *τ*_2_ values for the EPSC in Figure 2d are 1.07 and 76.7 s, respectively, and the fitted *τ*_1_ and *τ*_2_ for the IPSC in Figure 2e are 11.2 and 145.3 s, respectively. Furthermore, the *I*_ds_ amplitude can be continuously adjusted by changing the number of optical pulses (Figure 2f,g). Experimental results demonstrate that after applying the pulsed light stimulations (including both excitatory and inhibitory stimulations) to our device, the induced PPF agrees well with the results observed in biological synapses.

**Figure 3:**
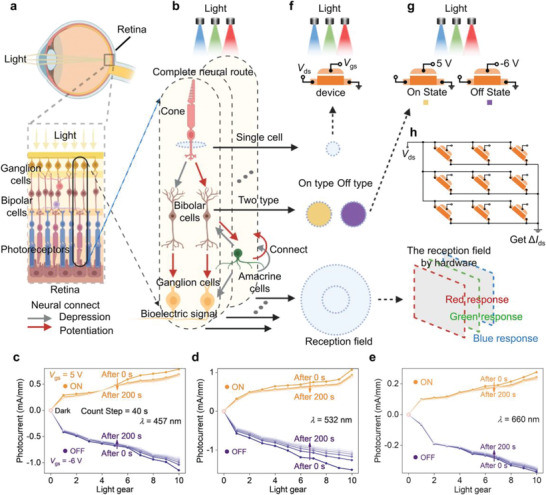
Simulation of retinal cells with HEMT‐based vision sensors. a) The structure of human retina. b) The process of light cross a complete neural route. Dependence of photocurrent on light gear with long retention time for c) 457 nm, d) 532 nm, and e) 660 nm laser illumination, respectively. f) A HEMT‐based vision sensor used to mimic single cone cell. g) HEMT‐based vision sensors operating at different gate voltage to simulate bipolar cell. h) The structure of the hardware kernel used to simulate different receptive field. Figure 3a, b and c are created with BioRender.com.

Next, we perform simulation to demonstrate the feasibility of the fabricated visual sensor in NVS. In human visual system, retina converts visual information to neural pulses, and then transmits them to brain through optic nerves and synapses. As shown in **Figure** **3**a, the structure of the retina is roughly grouped into three layers:^[^ ^44^, ^45^ ^]^ photoreceptor layer (PRL), bipolar cell layer (BCL), and ganglion cell layer (GCL). The complete optic neural route (ONR) in the retina consists of photoreceptor cells‐bipolar cells‐ganglion cells connectivity (Figure 3b). Specifically, the conversion of optical signals into electrical signals occurs inside the photoreceptor cells, which are composed of rod cells and cone cells. Cone cells can process color information, and rod cells are sensitive to incident light intensity. Like photoreceptors, our HEMT‐based visual sensor is also sensitive to wavelength and intensity of incident light (Figure S8, Supporting Information). Here, we define the value of the light gear (1–10) to map the light intensity (1–300 mW cm^−2^). The photocurrent under different light gear and wavelength extracted from Figure S8 (Supporting Information) is shown in Figure 3c–e, which demonstrates that the presented device is capable of simulating the biological behavior of photoreceptor cells (Figure 3f). In addition, bipolar cells that receive cone cells input are further divided into ON type and OFF type. ON type cells would hyperpolarize when they receive cone cell signals, while OFF type cells would depolarize. The behavior of hyperpolarization and depolarization can be simply understood as the inhibition and excitation of current when the visual sensors are operated under different gate voltages (Figure 3g). And then, the two types of bipolar cells transmit information to ganglion cells that can generate action potentials.^[^ ^44^ ^]^ The output of ganglion cells is not only determined by incident light, but also by the structure of the ONR when stimulated by light. Therefore, the concept of “receptive field” (RF) needs to be introduced. To maximize the output rate of ON‐type cells, it is necessary to give light in the middle area of the receptive field and withdraw light in the surrounding area, we defined this as ON‐RF; on the contrary, the output rate of OFF‐type cells is the highest when the light is withdrawn in the middle of the receptive field and the surrounding area is given light, we defined this as OFF‐RF. According to the above‐mentioned operation principle, the hardware kernel is constructed by integrating 9 HEMT‐based visual sensors, which can be separately controlled by gate voltage, as shown in Figure 3h. The output (*I*_output_) of hardware kernel is a summation of photocurrent from all devices (ΣΔ𝐼), and the real‐time variations of *I*_output_ corresponds to the dynamic change of the input light signal. Here, the same hardware kernel would induce different *I*_output_ values upon red, green, and blue light stimulus.

**Figure 4:**
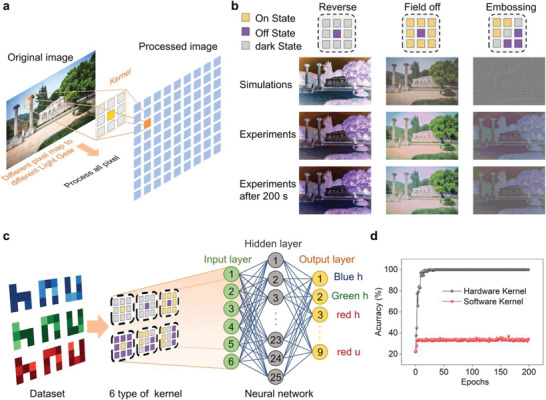
The image preprocessing and classification using a HEMT‐based artificial NVS. a) Schematic diagram of an artificial NVS based on the HEMT‐based vision sensors. b) The image preprocessing under three operations (“reverse,” “OFF‐RF,” and “embossing”). c) Schematic of the color image recognition based on the proposed NVS. The randomly generated training‐testing dataset consist of 9 types of letters, and the neural network is constructed by 6 input neurons, 25 hidden neurons, and 9 output neurons. d) Image recognition accuracy based on the hardware and software kernels, respectively.

The pivotal role of the receptive field in human retina is to accelerate the visual perception by visual information preprocessing. As shown in **Figure** **4**a, the key features of an image can be extracted by a 3 × 3 hardware kernel, and Figure 4b exhibits the preprocessing results using three different hardware kernels: “Reverse,” “OFF‐RF,” and “Embossing” stylization. Detailed information of image processing is given Figure S9 (Supporting Information). Here, the stylized images using the hardware kernels are similar to the computer simulation results. The images can be reliably memorized for 200 s due to the excellent optical storage characteristic of the visual sensors. Furthermore, a NVS composed of artificial neural network (ANN) and HEMT‐based visual sensor array is designed for color object identification. The sensor array is employed as the preprocessing part to extract key features of the image, while the ANN can recognize the information provided by the former. As shown in Figure 4c, the object is made of 9 types of color images (3 × 3 pixels), including letters (“h,” “n,” and “u”) in RGB (red, green, and blue) colors. The pixel value of the image is defined in the range of 0 (colorless) to 1 (colorfully). In the experiments, 3600 images are randomly generated to train the ANN, and another 900 images are used for identification testing. The neural network model defined in this paper contains three layers: input layer, hidden layer, and output layer. There are 6 neurons in the input layer, corresponding to 6 key feature values extracted by the kernels shown in Figure 4c. Notably, the key feature values output by the hardware kernels are based on the photoresponse in Figure 3c–e, and the values of the light gear that determines the magnitude of the photocurrent are obtained by mapping the pixel values of the image. Meanwhile, during the algorithmic process of the three‐layer ANN 6 × 25 × 9, sigmoid function is adopted as activation function.^[^ ^46^ ^]^ As shown in Figure 4d, the recognition accuracy is close to 100% with no more than 50 epochs, which is much better than that of the dataset using the software kernel (32%). The successful functional verifications of neuromorphic image preprocessing and color recognition demonstrate that the proposed HEMT‐based visual sensor is suitable for the complex neuromorphic visual system.

## 3 Conclusion

In summary, we have designed and fabricated a highly sensitive visual sensor based on 2D OIHP‐gated D‐mode AlGaN/GaN HEMT, which can respond to visible light stimuli directly with a nonvolatile manner. The cryogenic electrical experiments confirm the field‐effect‐based photoresponse mechanism. Light‐dependent ion transport in 2D OIHP film is quantitatively evaluated by extracting ion conductivity over a variety of illumination intensity, which exerts a significant impact on the optical memory function. The bidirectional photoresponse is achieved by switching *V*_gs_ polarity following light‐modulated synaptic behavior, such as EPSC, IPSC, and PPF. Besides, the proposed sensor can be programmed to simultaneously sense and preprocess images with distinct kernels. Moreover, a NVS is constructed by the sensor array, and the recognition accuracy of color images is improved from 32% to 100% through the hardware kernel process. The results demonstrate the feasibility of large‐scale deployment of AlGaN/GaN HEMTs in the emerging neuromorphic computing.

## 4 Experimental Section

### Device Fabrication

The AlGaN/GaN heterostructures were grown on SiC substrate by MOCVD system. From bottom to top, the growing vertical heterostructures were composed of 100 nm AlN nucleation layer, 1.5 µm GaN buffer layer, 1 nm AlN interlayer, and 22 nm AlGaN barrier layer respectively. Subsequently, source/drain electrodes were obtained by Ni/Au metal thermal evaporation. The channel length was 100 µm and the channel width was 200 µm. Then, a 12‐nm HfO_2_ film was deposited by ALD at 95 °C, and the growth rate of the HfO_2_ film was 1.2 Å per cycle. In order to prepare precursor solution of 2D OIHP (PEA)_2_PbI_4_, specific stoichiometric quantities PEAI and PbI_2_ were dissolved in anhydrous *N*, *N*‐dimethylformamide solvent in a nitrogen‐filled glovebox. The mixed solution was placed on a hot plate at 50 °C and stirred continuously for 8 h. The obtained solution was spin‐coated on HfO_2_ layer at 3500 rpm for 60 s, and the resulting sample was annealed on a hot plate at 100 °C for 60 min. Finally, the Al (25 nm) gate electrode was deposited on 2D OIHP film by metal evaporation process through a shadow mask.

### Material Characterization and Device Measurement

The morphology of 2D OIHP film was characterized by SEM (JEOL 6510). XRD measurement was carried out by employing the X‐ray Diffractometer (Rigaku SmartLab). The absorption spectrum was characterized by the UV−vis spectrometer (Shimadzu UV‐2550). The optoelectronic properties of the HEMT‐based visual sensor were measured by the semiconductor parameter analyzer (Agilent B1500A). The low‐temperature electrical measurements were performed by the Lake Shore TTPX. The incident light sources were lasers with wavelengths of 375, 457, 532, 660, and 808 nm, respectively.

### Simulation Method

All the part of the retina‐neuromorphic vision sensor was mimicked by Python program, and all the data in the program was came from the reality experiment metadata of the HEMTs‐based vision sensor.

## Conflict of Interest

The authors declare no conflict of interest.

## Supporting information

Supporting Information

## Data Availability

The data that support the findings of this study are available from the corresponding author upon reasonable request.

## References

[1] S. Park , M. Chu , J. Kim , J. Noh , M. Jeon , B. H. Lee , H. Hwang , B. Lee , B. Lee , Sci. Rep. 2015, 5, 10123. 25941950 10.1038/srep10123 PMC4419523

[2] P. A. Merolla , J. V. Arthur , R. Alvarez‐Icaza , A. S. Cassidy , J. Sawada , F. Akopyan , B. L. Jackson , N. Imam , C. Guo , Y. Nakamura , B. Brezzo , I. Vo , S. K. Esser , R. Appuswamy , B. Taba , A. Amir , M. D. Flickner , W. P. Risk , R. Manohar , D. S. Modha , Science 2014, 345, 668. 25104385 10.1126/science.1254642

[3] K. Lee , H. Han , Y. Kim , J. Park , S. Jang , H. Lee , S. W. Lee , H. Y. Kim , Y. Kim , T. Kim , D. Kim , G. Wang , C. Park , Adv. Funct. Mater. 2021, 31, 2105596.

[4] F. Zhou , Z. Zhou , J. Chen , T. H. Choy , J. Wang , N. Zhang , Z. Lin , S. Yu , J. Kang , H. P. Wong , Y. Chai , Nat. Nanotechnol. 2019, 14, 776. 31308498 10.1038/s41565-019-0501-3

[5] S. Seo , S. H. Jo , S. Kim , J. Shim , S. Oh , J.‐H. Kim , K. Heo , J.‐W. Choi , C. Choi , S. Oh , D. Kuzum , H. S. P. Wong , J.‐H. Park , Nat. Commun. 2018, 9, 5106. 30504804 10.1038/s41467-018-07572-5 PMC6269540

[6] J. Du , D. Xie , Q. Zhang , H. Zhong , F. Meng , X. Fu , Q. Sun , H. Ni , T. Li , E. Guo , H. Guo , M. He , C. Wang , L. Gua , X. Xu , G. Zhang , G. Yang , K. Jin , C. Ge , Nano Energy 2021, 89, 106439.

[7] Y. X. Hou , Y. Li , Z. C. Zhang , J. Q. Li , D. H. Qi , X. D. Chen , J. J. Wang , B. W. Yao , M. X. Yu , T. B. Lu , J. Zhang , ACS Nano 2021, 15, 1497. 33372769 10.1021/acsnano.0c08921

[8] C. Y. Wang , S. J. Liang , S. Wang , P. Wang , Z. A. Li , Z. Wang , A. Gao , C. Pan , C. Liu , J. Liu , H. Yang , X. Liu , W. Song , C. Wang , X. Wang , K. Chen , Z. Wang , K. Watanabe , T. Taniguchi , J. J. Yang , F. Miao , Sci. Adv. 2020, 6, 6173.

[9] X. Han , Z. Xu , W. Wu , X. Liu , P. Yan , C. Pan , Small Struct. 2020, 1, 2000029.

[10] F. Y. Liao , F. C. Zhou , Y. Chai , J. Semicond. 2021, 42, 013105.

[11] B. Pradhan , S. Das , J. Li , F. Chowdhury , J. Cherusseri , D. Pandey , D. Dev , A. Krishnaprasad , E. Barrios , A. Towers , A. Gesquiere , L. Tetard , T. Roy , J. Thomas , Sci. Adv. 2020, 6, 5225. 10.1126/sciadv.aay5225 PMC7015692 32095529

[12] S. Chen , Z. Lou , D. Chen , G. Shen , Adv. Mater. 2018, 30, 1705400. 10.1002/adma.201705400 29315837

[13] M. A. Zidan , J. P. Strachan , W. D. Lu , Nat. Electron. 2018, 1, 22.

[14] S. E. Ng , J. Yang , R. A. John , N. Mathews , Adv. Funct. Mater. 2021, 31, 2100807.

[15] Z. Q. Wang , H. Y. Xu , X. H. Li , H. Yu , Y. C. Liu , X. J. Zhu , Adv. Funct. Mater. 2012, 22, 2759.

[16] L. Tong , Z. Peng , R. Lin , Z. Li , Y. Wang , X. Huang , K. Xue , H. Xu , F. Liu , H. Xia , P. Wang , M. Xu , W. Xiong , W. Hu , J. Xu , X. Zhang , L. Ye , X. Miao , Science 2021, 373, 1353. 34413170 10.1126/science.abg3161

[17] F. Xue , X. He , Z. Wang , J. Retamal , Z. Chai , L. Jing , C. Zhang , H. Fang , Y. Chai , T. Jiang , W. Zhang , H. Alshareef , Z. Ji , L. Li , J. He , X. Zhang , Adv. Mater. 2021, 33, 2008709. 10.1002/adma.202008709 33860581

[18] Y. Wang , Y. Gong , L. Yang , Z. Xiong , Z. Lv , X. Xing , Y. Zhou , B. Zhang , C. Su , Q. Liao , S. Han , Adv. Funct. Mater. 2021, 31, 2100144.

[19] X. Yan , Q. Zhao , A. Chen , J. Zhao , Z. Zhou , J. Wang , H. Wang , L. Zhang , X. Li , Z. Xiao , K. Wang , C. Qin , G. Wang , Y. Pei , H. Li , D. Ren , J. Chen , Q. Liu , Small 2019, 15, 1901423.

[20] X. Yan , L. Zhang , H. Chen , X. Li , J. Wang , Q. Liu , C. Lu , J. Chen , H. Wu , P. Zhou , Adv. Funct. Mater. 2018, 28, 1803728.

[21] C. Wu , T. W. Kim , H. Y. Choi , D. B. Strukov , J. J. Yang , Nat. Commun. 2017, 8, 752. 28963546 10.1038/s41467-017-00803-1 PMC5622032

[22] W. Xu , H. Cho , Y. H. Kim , Y. T. Kim , C. Wolf , C. G. Park , T. W. Lee , Adv. Mater. 2016, 28, 5916. 27167384 10.1002/adma.201506363

[23] H. Han , H. Yu , H. Wei , J. Gong , W. Xu , Small 2019, 15, 1900695. 10.1002/smll.201900695 30972944

[24] Q. Wang , Y. Wen , K. Cai , R. Cheng , L. Yin , Y. Zhang , J. Li , Z. Wang , F. Wang , F. Wang , T. A. Shifa , C. Jiang , H. Yang , J. He , Sci. Adv. 2018, 4, 7916. 10.1126/sciadv.aap7916 PMC5954648 29770356

[25] J. Yu , X. Yang , G. Gao , Y. Xiong , Y. Wang , J. Han , Y. Chen , H. Zhang , Q. Sun , Z. L. Wang , Sci. Adv. 2021, 7, 9117. 10.1126/sciadv.abd9117 PMC7968845 33731346

[26] L. Shao , H. Wang , Y. Yang , Y. He , Y. Tang , H. Fang , J. Zhao , H. Xiao , K. Liang , M. Wei , W. Xu , M. Luo , Q. Wan , W. Hu , T. Gao , Z. Cui , ACS Appl. Mater. Interfaces 2019, 11, 12161. 30817113 10.1021/acsami.9b02086

[27] H. Tian , Q. Guo , Y. Xie , H. Zhao , C. Li , J. J. Cha , F. Xia , H. Wang , Adv. Mater. 2016, 28, 4991. 27119423 10.1002/adma.201600166

[28] H. Tian , X. Cao , Y. Xie , X. Yan , A. Kostelec , D. DiMarzio , C. Chang , L. Zhao , W. Wu , J. Tice , J. J. Cha , J. Guo , H. Wang , ACS Nano 2017, 11, 7156. 28656774 10.1021/acsnano.7b03033

[29] M. Khaouani , A. Hamdoune , H. Bencherif , Z. Kourdi , L. Dehimi , Optik 2020, 217, 164797.

[30] Q. Lyu , H. Jiang , K. M. Lau , Appl. Phys. Lett. 2020, 117, 071101.

[31] J. Z. Li , J. Y. Lin , H. X. Jiang , M. A. Khan , Appl. Phys. Lett. 1998, 72, 2868.

[32] A. Gundimeda , S. Krishna , N. Aggarwal , A. Sharma , N. D. Sharma , K. K. Maurya , S. Husale , G. Gupta , Appl. Phys. Lett. 2017, 110, 103507.

[33] T. M. Kuan , S. J. Chang , Y. K. Su , C. H. Ko , J. B. Webb , J. A. Bardwell , Y. Liu , H. Tang , W. J. Lin , Y. T. Cherng , W. H. Lan , Jpn. J. Appl. Phys. 2003, 42, 5563.

[34] M. Hou , H. So , A. J. Suria , A. S. Yalamarthy , D. G. Senesky , IEEE Electron Device Lett. 2017, 38, 56.

[35] M. H. Li , H. H. Yeh , Y. H. Chiang , U. S. Jeng , C. J. Su , H. W. Shiu , Y. J. Hsu , N. Kosugi , T. Ohigashi , Y. A. Chen , P. S. Shen , P. Chen , T. F. Guo , Adv. Mater. 2018, 30, 1801401. 10.1002/adma.201801401 29883002

[36] J. Hao , Y. H. Kim , S. N. Habisreutinger , S. P. Harvey , E. M. Miller , S. M. Foradori , M. S. Arnold , Z. Song , Y. Yan , J. M. Luther , J. L. Blackburn , Sci. Adv. 2021, 7, 1959. 10.1126/sciadv.abf1959 PMC8081365 33910894

[37] D. Li , H. Wu , H.‐C. Cheng , G. Wang , Y. Huang , X. Duan , ACS Nano 2016, 10, 6933. 27315525 10.1021/acsnano.6b02795

[38] C. Xie , P. You , Z. Liu , L. Li , F. Yan , Light: Sci. Appl. 2017, 6, e17023. 30167278 10.1038/lsa.2017.23 PMC6062319

[39] Y. C. Zhao , W. K. Zhou , X. Zhou , K. H. Liu , D. P. Yu , Q. Zhao , Light: Sci. Appl. 2017, 6, e16243. 30167249 10.1038/lsa.2016.243 PMC6062189

[40] J. Mizusaki , Arai , K. Fueki , Solid State Ionics 1983, 11, 203.

[41] Y. T. Li , L. Ding , J. Z. Li , J. Kang , D. H. Li , L. Ren , Z. Y. Ju , M. X. Sun , J. Q. Ma , Y. Tian , G. Y. Gou , D. Xie , H. Tian , Y. Yang , L. W. Wang , L. M. Peng , T. L. Ren , ACS Cent. Sci. 2019, 5, 1857. 31807687 10.1021/acscentsci.9b00839 PMC6891861

[42] Q. Tian , R. Hong , C. Liu , X. Hong , S. Zhang , L. Wang , Y. Lv , X. Liu , X. Zou , L. Liao , Nano Lett. 2022, 22, 494. 34964627 10.1021/acs.nanolett.1c04402

[43] S. Gao , G. Liu , H. Yang , C. Hu , Q. Chen , G. Gong , W. Xue , X. Yi , J. Shang , R. W. Li , ACS Nano 2019, 13, 2634. 30730696 10.1021/acsnano.9b00340

[44] C. Joselevitch , Psychol. Neurosci. 2008, 1, 141.

[45] C. A. Curcio , K. A. Allen , J. Comp. Neurol. 1990, 300, 5. 2229487 10.1002/cne.903000103

[46] J. Han , C. Moraga , International Workshop on Artificial Neural Networks, Springer, Berlin, Heidelberg 1995, p. 195.

