# Supplementary material for: Two‐Dimensional Perovskite‐Gated AlGaN/GaN High‐Electron‐Mobility‐Transistor for Neuromorphic Vision Sensor
Source: Adv Sci (Weinh). 2022 Jul 22;9(27):2202019. doi: 10.1002/advs.202202019 (PMC9507368; doi:10.1002/advs.202202019)
Supplement: Supplementary file 1 — Supporting Information [file ADVS-9-2202019-s001.pdf]

Supporting information

Two-dimensional Perovskite-gated AlGaN/GaN High-electron-mobility-transistor for Neuromorphic Vision Sensor

Xitong Hong, Yulong Huang, Qianlei Tian, Sen Zhang, Chang Liu, Liming Wang, Kai Zhang, Jia Sun, Lei Liao*, Xuming Zou**

Figure S1. Characterization of $(PEA)_2PbI_4$ film.

Figure S2. Gate leakage curves of the HEMT-based visual sensor.

Figure S3. Optoelectronic characteristics of the HEMT-based visual sensor.

Figure S4. Optoelectronic characteristics of the AlGaN/GaN HEMT.

Figure S5. Evaluation of ionic conductivity at different temperature and illumination intensity.

Figure S6. Storage and erasing characteristics of the HEMT-based visual sensor.

Figure S7. Proposed photoresponse mechanism of the HEMTs-based visual sensor.

Figure S8. I_{ph}-T curves of the HEMT-based visual sensor upon different incident light.

Figure S9. The details of the image processing by hardware kernel.

Supplementary Figures:

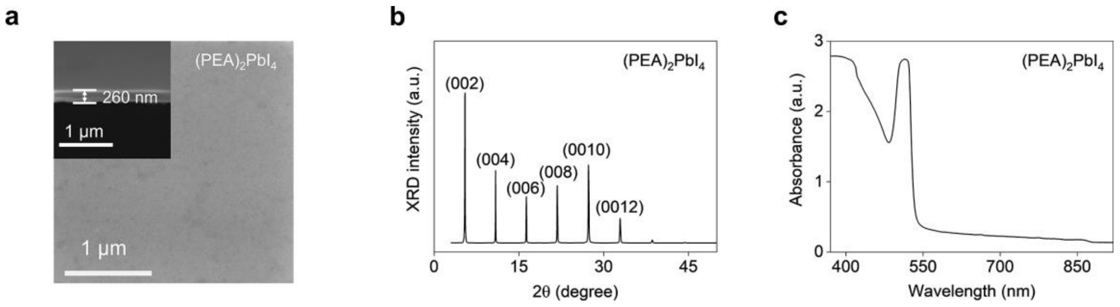

Figure S1. Characterization of $(PEA)_2PbI_4$ film. (**a**) A typical SEM image of the $(PEA)_2PbI_4$ layer deposited on HfO_2 film. The inset shows the corresponding cross-sectional SEM image. (**b**) XRD spectrum of the $(PEA)_2PbI_4$ perovskite film. The strong periodic peaks located at 5.5°, 11°, 16.6°, 22°, 27.5°, and 33°, which can be indexed as (002), (004), (006), (008), (0010), and (0012) planes respectively. (**c**) The absorption spectrum of the 2D OIHP $(PEA)_2PbI_4$ film with a remarkable peak at 515 nm.

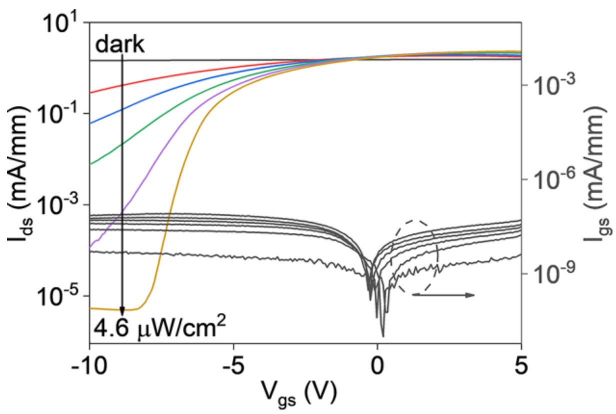

Figure S2. Gate leakage curves of the HEMT-based visual sensor under different laser power density at V_{ds} = 0.1 V.

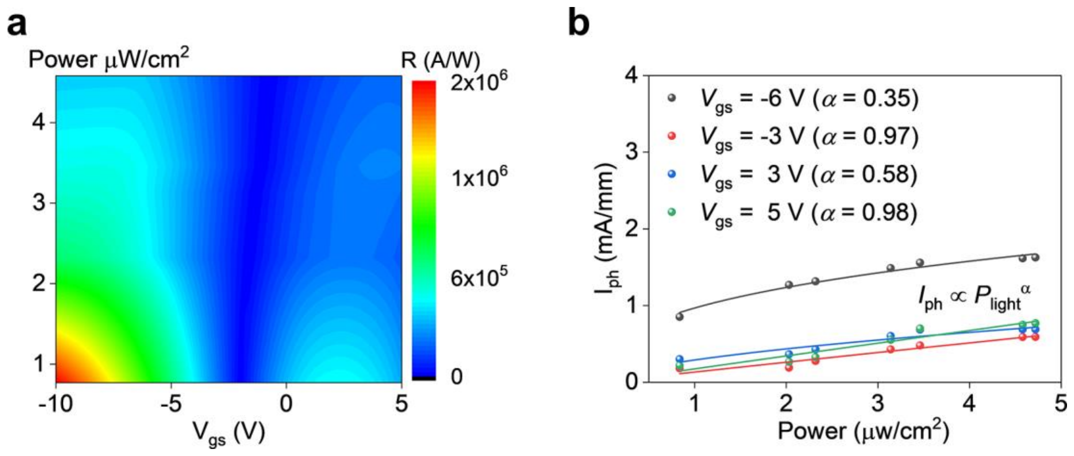

Figure S3. Optoelectronic characteristics of the HEMT-based visual sensor. (**a**) V_{gs} and P_{light} dependent photoresponsivity extracted from transfer characteristic curves in Figure 1c. (**b**) The I_{ph} values plotted as a function of incident power at different gate voltage.

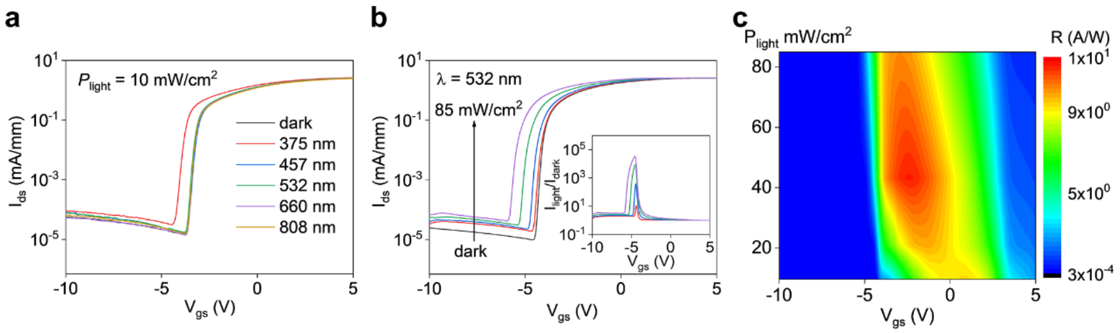

Figure S4. Optoelectronic characteristics of the AlGaN/GaN HEMT. (a) Transfer characteristic curves of the AlGaN/GaN HEMT upon different wavelength laser illumination. (b) Transfer characteristic of the GaN HEMT measured under various illumination intensity. The inset depicts the light-to-dark current ratio under different P_{light} values. (c) V_{gs} and P_{light} dependent photoresponsivity extracted from transfer characteristic curves in Figure S4(b).

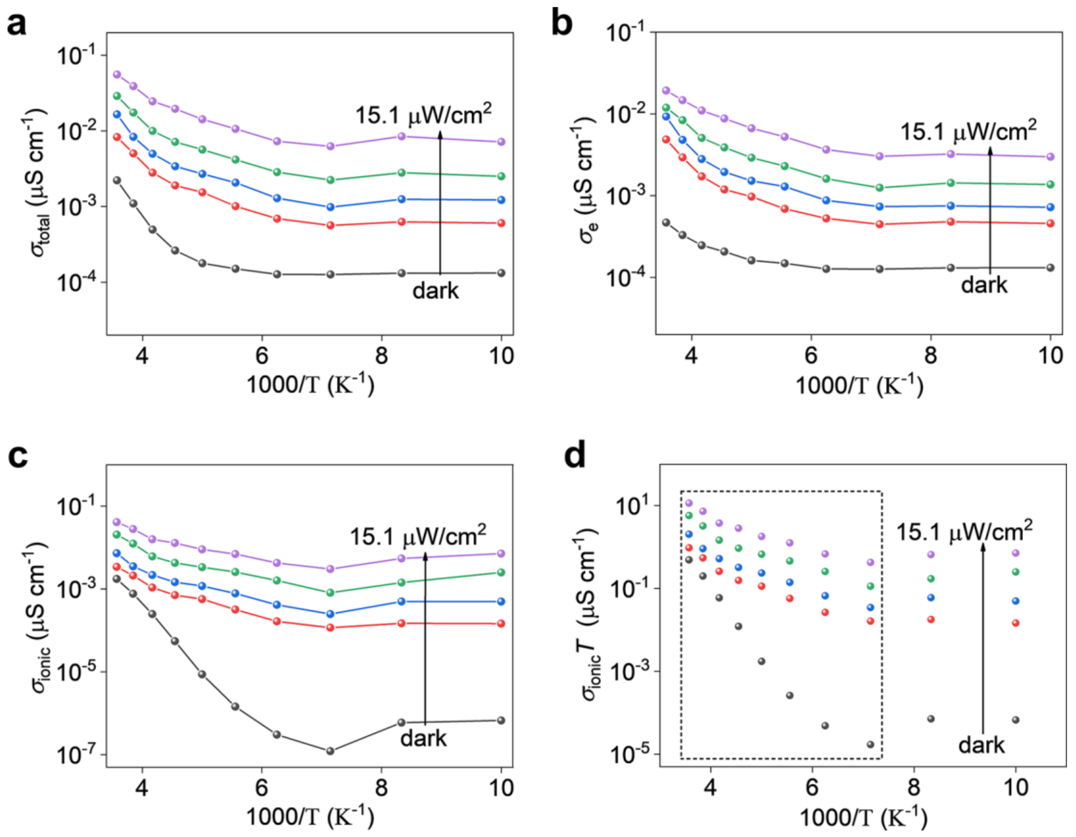

Figure S5. Evaluation of ionic conductivity at different temperature and illumination intensity. (**a**) Mixed conductivity at different temperature from 100 to 295 K upon various illumination intensity. (**b**) Electronic conductivity at different temperature from 100 to 295 K upon various illumination intensity. (**c**) Ionic conductivity at different temperature from 100 to 295 K upon various illumination intensity. (**d**) The $\sigma_{ion}T$ values at different temperature from 100 to 295 K upon various illumination intensity. The zoomed-in view of the linear region is shown in Figure 1h.

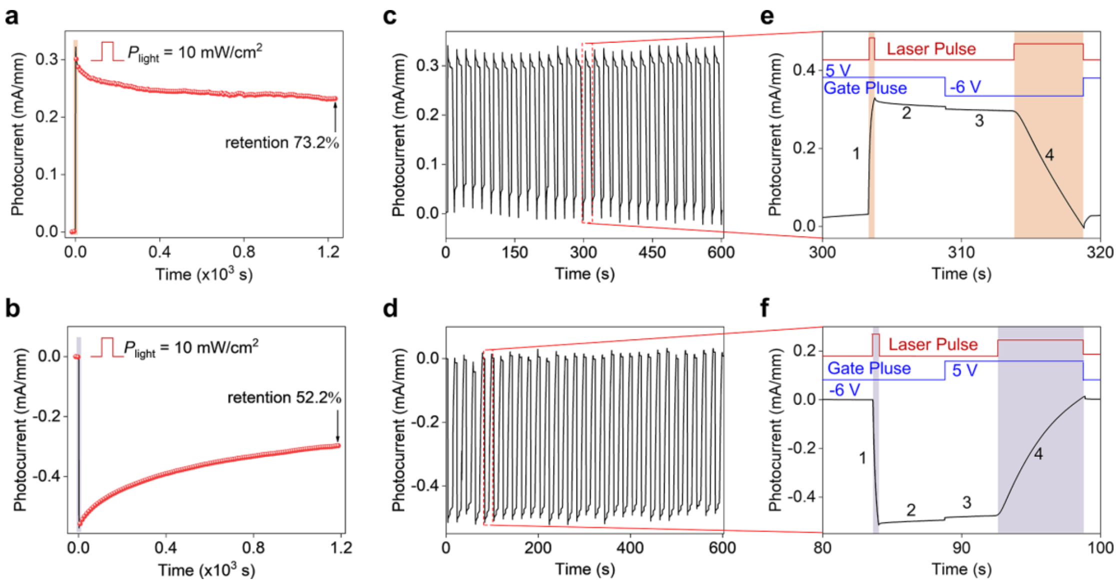

Figure S6. Storage and erasing characteristics of the HEMT-based visual sensor. Time-dependent photocurrent of the device stimulated by 532 nm laser pulse (10 mW/cm^2, 1 s) under V_{gs} of 5 V (**a**) and -6 V (**b**). Multiple cycles of programing and erasing processes of the device at V_{gs} = 5 V (**c**) and V_{gs} = -6 V (**d**). (**e,f**) A single operation cycle. Here, the programmed state cannot be completely erased by V_{gs} pulse alone. The recovery of the initial state is achieved by applying V_{gs} on the device with light illumination.

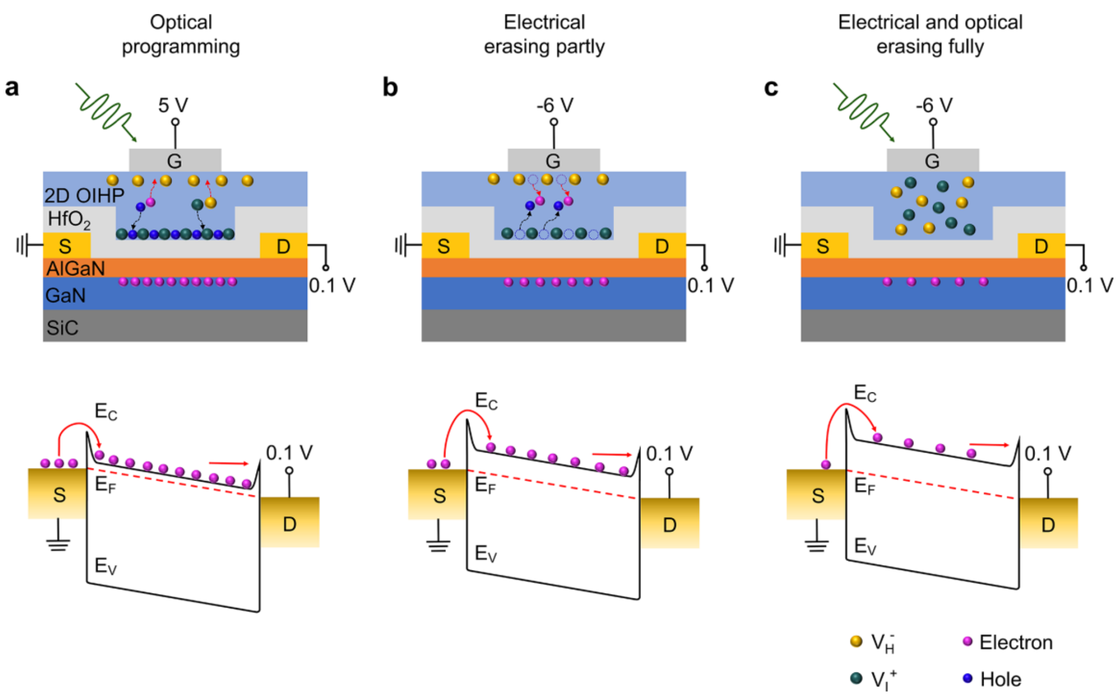

Figure S7. Proposed photoresponse mechanism of the HEMTs-based visual sensor. Schematic diagram showing the influence of electrons/holes and vacancies drift on band energy of the HEMTs-based visual sensor under optical programming process (**a**), partly erasing process using V_{gs} pulse (**b**), and fully erasing process using V_{gs} and light pulses (**c**). E_C, E_V, and E_F denote the conduction band edge, valence band edge, and Fermi level, respectively.

Here, we take the positive photoresponse as an example. By applying a positive gate voltage of 5 V with light illumination, photo-excited holes and cations are accumulated at the 2D OIHP/HfO_2 interface, which increases the 2DEG concentration and leads to a positive photocurrent (Figure S7a). For erasing, a negative gate voltage of -6 V is applied under dark. In this case, the trapped holes would be released via the recombination with the injected electrons. However, it is difficult to realize the fully erasing operation by applying negative gate voltage alone, which is probably due to the enlarged energy barrier for ion transport in 2D OIHP under dark (Figure S7b). In comparison, under a negative gate voltage of -6 V with light illumination, the accumulated ions can move away from the interfaces with time. Once all ions return to the equilibrium state, the electric field induced by the ions would be reduced to zero, resulting in a zero photocurrent (Figure S7c).

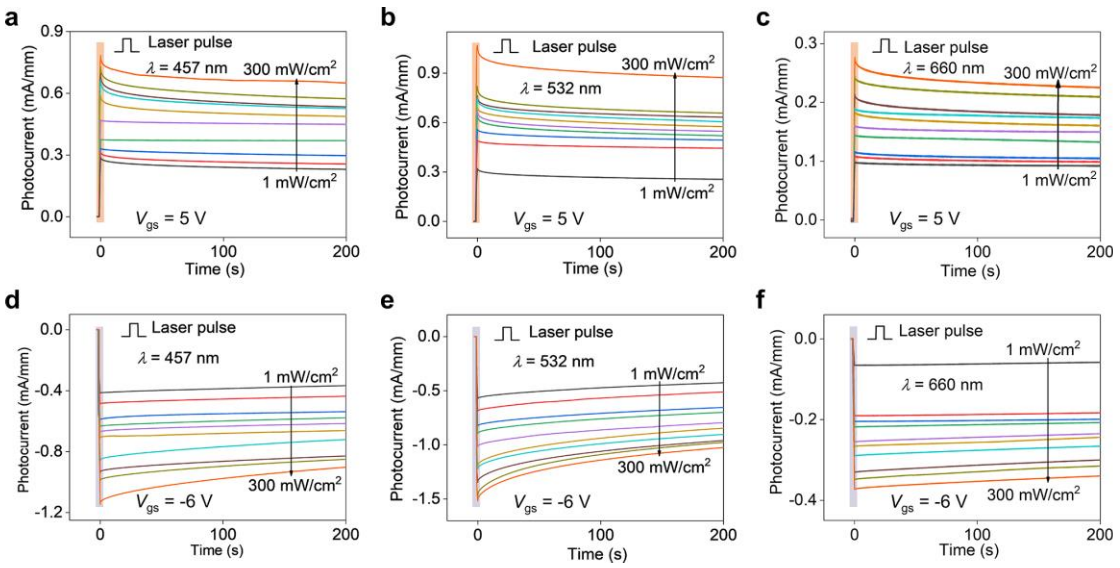

Figure S8. I_{ph}-T curves of the HEMT-based visual sensor upon different incident light. Time-dependent I_{ph} of the HEMT-based visual sensor illuminated with different wavelength (457 nm, 532 nm, 660 nm) of incident light at V_{gs} = 5 V (**a-c**) and V_{gs} = -6 V (**d-f**), and the laser duration time is 1 s with varying light intensity from 1 to 300 mW/cm^2.

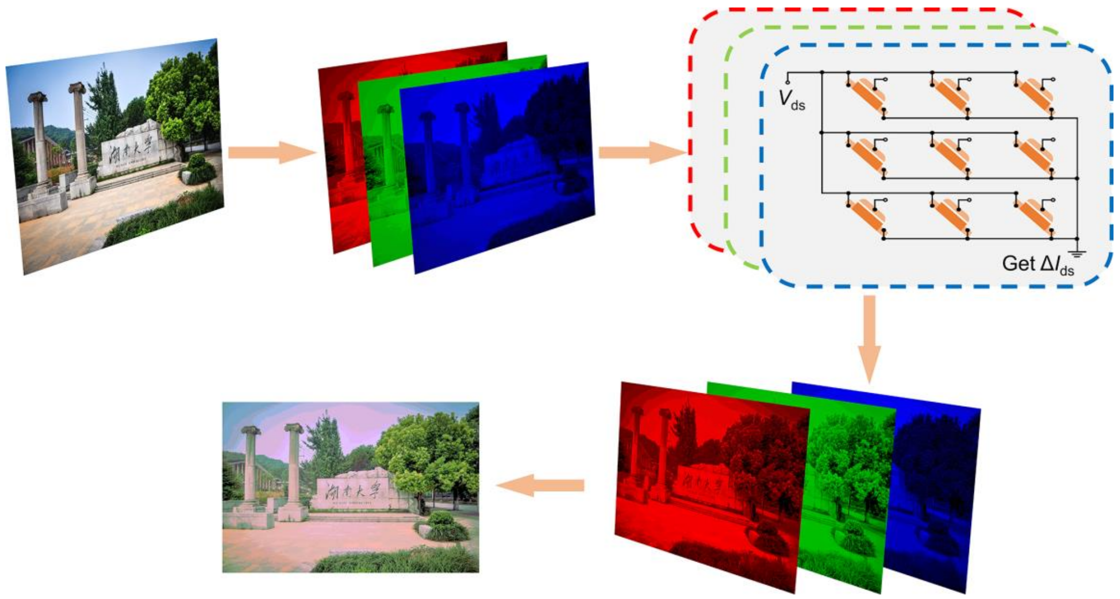

Figure S9. The details of the image processing by hardware kernel.

Here, we emulated the receptive field by integrating 9 HEMT-based vision sensors separately controlled by the gate voltage into an array, and the process images using hardware kernel is as follows. Firstly, a color image is divided into three color channels (R, G and B), which are mapped to the corresponding light wavelength (660/532/457 nm) in Figure 3c-3e. Then, the pixel values (0-255) of each channel are normalized (0-1) and divided into 10 gears with the interval of 0.1. Finally, the three color channels (R, G, B) are combined to form a new color image.
